# Stress-Strain Response of Cylindrical Rubber Fender under Monotonic and Cyclic Compression

**DOI:** 10.3390/ma12020282

**Published:** 2019-01-16

**Authors:** Chia-Chin Wu, Yung-Chuan Chiou

**Affiliations:** 1 Department of Mechanical and Energy Engineering, National Chiayi University, Chiayi 60004, Taiwan; joechia-chin.wu@mail.ncyu.edu.tw; 2 Department of Biomechatronic Engineering, National Chiayi University, Chiayi 60004, Taiwan

**Keywords:** rubber fender, monotonic/cyclic behavior, energy/stress polynomials

## Abstract

The study was devoted to the observation and modeling the mechanical behaviors of a hybrid SBR/NR (Styrene-Butadiene/Natural Rubber) hybrid vulcanized rubber fender under monotonic/cyclic compression. In experimental observations of the monotonic compression tests, it was found that lateral deformation occurred on the tested fender and was more significant with increasing the extent of the compressive strain. The relationship between the transmission stress $S_{c}$ and the compressive strain $e_{c}$ was nonlinear and the absorbed strain-energy-density was increased monotonically with the increment of the compressive strain. Among all cyclic compression tests with strain controlled, the reductions in both the stress range and the absorbed strain-energy-density up to the ten-thousandth cycle were found and then both of the cyclic properties remain approximately constant in the following compression cycles. Two new properties, the softening factor and the energy reduction factor, were introduced to quantify the effect of the strain range on the extent of the reduction in stress range and that on the absorbed strain-energy-density, respectively. It was found that both of the calculated values of the new properties increase with the increment of strain range. In mathematical modeling of the relationship between the transmission stress and the compressive strain, a new approach based on energy-polynomial-function $E_{s}\left(e_{c}\right)$ was presented and was successfully used to simulate the monotonic curve and the stable hysteresis loop curves of the tested rubber fender in compression. Essentially, the energy-polynomial-function $E_{s}\left(e_{c}\right)$ was obtained by performing a polynomial regression on a large amount of ($e_{c},E_{s})$ data. Moreover, the least-square approach was applied to determine the corresponding regression coefficients in $E_{s}\left(e_{c}\right)$. Clearly, the stress-polynomial-function in modeling the $S_{c}-e_{c}$ curve could be obtained from the differentiation of the energy-polynomial-function with respect to the compressive strain. In addition, to provide an adequate estimation of the mechanical properties of the cylindrical rubber fender under compression, the named cyclic stress-strain curve and cyclic energy-strain curve were developed and also modeled in this study.

## 1. Introduction

Marine fender is a component installed at the dock to reduce and/or prevent damage when the vessel is berthing at a certain speed and hitting the fender. In the event of berthing, the kinetic energy of the vessel is absorbed by the compressive deformation of the fender and the accompanying water with the vessel, thereby reducing the risk of destruction of both vessel and dock. An effective fender requires both good mechanical durability and excellent energy absorption. The rubber fender studied in this paper is cylindrical, and its energy absorption is the key consideration. Since rubber materials provide large elastic strain in compression and excellent energy absorption capability, they are one of the most commonly used damping mechanisms in practical engineering applications [1,2] where the energy absorption is a key consideration. 

Rubber components are made from one of the three different raw materials, namely the natural rubber (NR), the synthetic rubber, and the hybrid rubber, where the choice of raw material depends on the functional requirements of the particular application, such as temperature, frequency, deformation, and the environment. Hence, numerous experimental studies [3,4,5,6,7,8,9,10,11,12,13] investigate the mechanical behavior of rubber materials. It is noted that the three kinds of synthetic rubber, the styrene-butadiene (SBR), the chloroprene rubber (CR, neoprene), and the ethylene-propylene-diene monomer (EPDM) have wide ranges of application in sensors, components, and devices. Moreover, hybrid rubber materials, which is combined with both the NR and the synthetic rubber (e.g., SBR, CR), provide both excellent energy-absorption capability and good environmental resistance [14]. Hence, the hybrid rubber materials are commonly used to manufacture rubber components, such as tires, rubber roller, rubber fender, and so on. Notably, the SBR/NR (Styrene-Butadiene/Natural Rubber) hybrid vulcanized rubber is usually used to make the rubber fender in Taiwan because of the relatively strong sunlight. Since the mechanical behavior of the marine fender under different loading conditions are still not comprehensively understood, it is important to accurately model the mechanical behaviors for designing a new type of marine fender. Rubber fenders mounted at the dock are often subjected to compression loading, due to the berthing of the vessel. Therefore, this study focused on the mechanical behaviors of the cylindrical SBR/NR rubber fender under monotonic/cyclic compression and the variation of the measured transmission stress $S_{c}$ with the absorbed strain-energy-density $E_{s}$ under monotonic compression was firstly investigated. It was observed that the large lateral bending deformation occurred on the tested fender, which indicated that the buckling behavior of the tested fender dominated the ability of energy absorption. A nonlinear correlation was also found for both the developed $S_{c}-e_{c}$ curve and $E_{s}-e_{c}$ curve. In cyclic compression tests, it was found that the behavior of the lateral bending deformation became more obvious with increasing the extent of the strain range. Furthermore, it was found that the magnitude of the transmission stress range per cycle $\Delta S_{N}$ decreased with the increasing number of cycles (N). The phenomenon, which is known as Mullines’ effect [15], could be attributed to a fracture of the carbon black chains in the rubber material or a gradual weakening of the bonds between the rubber and the chains. The same behavior is reported in the studies [16,17]. Subsequently, the number of cycles corresponding to the approximately stable stress-strain response of the tested damper would be estimated from the experimental observations. To examine the extent of the change in magnitudes of $\Delta S_{N}$ and $E_{s,a}$ from the transient response to stable response at various cyclic strain levels, two new properties, the softening factor RS and the energy reduction factor $RE_{s,a}$, were presented in this study. Moreover, based on the experimental results of the monotonic compression tests, the energy-polynomial-function $E_{s}\left(e_{c}\right)$ expressed in terms of the variable $e_{c}$ was determined from performing a polynomial regression on a large amount of ($e_{c},\;E_{s})$ data. Similarly, the energy-polynomial-functions corresponding to the loading/unloading branches of the stable hysteresis-loop curve at various strain amplitudes were also developed. Besides, the stress functions $S_{c}\left(e_{c}\right)$ used to model the $S_{c}-e_{c}$ curve was obtained from the differentiation of the energy-polynomial-function $E_{s}\left(e_{c}\right)$ with respect to the compressive strain. In this study, those developed stress-polynomial-functions were respectively applied to model the $S_{c}-e_{c}$ curve in monotonic compression and the upper/lower branches of the stable hysteresis-loop curve under cyclic compression. The new approach in determining the stress-polynomial-functions $S_{c}\left(e_{c}\right)$ was verified by comparing the simulated results with the experimental measurements in this study. 

## 2. Experimental Procedure

### 2.1. Materials and Components 

**Figure 1:**
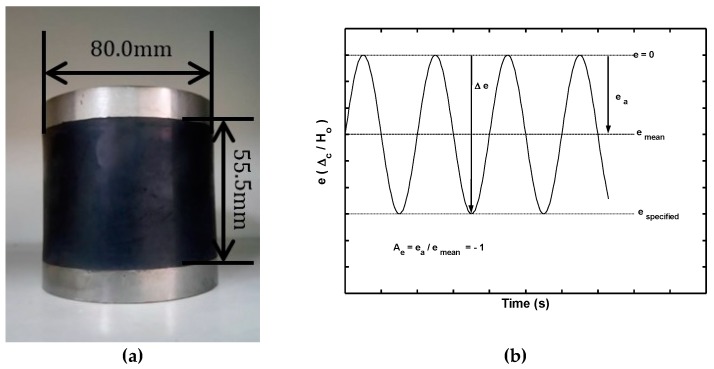

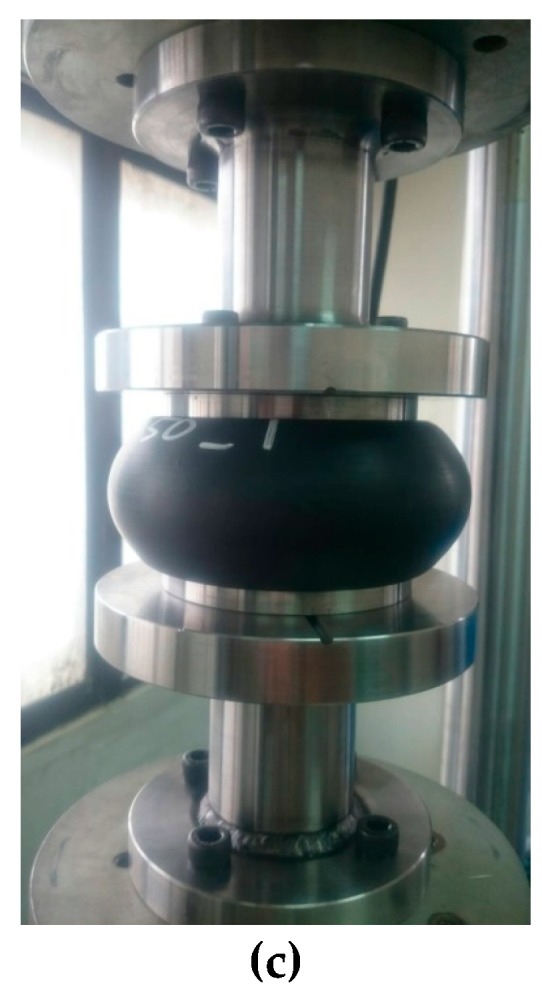
(**a**) Geometry and dimensions of tested rubber fender, (**b**) time history of cyclic strain signal, and (**c**) illustration of tested rubber fender at strain of −0.5 under monotonic compression.

The monotonic and cyclic compression tests were conducted by using rubber fenders with the geometry and dimensions, shown in Figure 1a. The fenders are purchased from Chin-Cheng Rubber Factory (C80ϕ, Chiayi, Taiwan) and are fabricated from SBR/NR vulcanized rubber with the composition summarized in Table 1. According to the manufacturer’s specification, the dampers have a hardness of Shore-A 66. 

### 2.2. Mechanical Testing 

To evaluate the deformation behavior of the fenders under realistic loading conditions, the transmission force response was measured under both the monotonic compression tests and the fully compressive cyclic tests with strain controlled. In both of the compression tests, the strain was defined as the ratio of the measured displacement in the loading direction, $\Delta_{c}$, to the original height of the fender, $H_{o}\;\left(i.e.,\;e_{c}=\Delta_{c}/H_{o}\right)$. Figure 1b showed the time history of the strain signal used in the cyclic compression tests, in which the strain varies between zero and a specified strain. In other words, the controlled mean strain level was equal to one-half of the specified strain, while the magnitude of the strain amplitude $e_{a}$ was equal to the absolute value of the controlled mean level strain, so that the amplitude ratio was given by $A_{e}=e_{a}/e_{mean}=-1$. 

In the monotonic compression tests, the rubber fenders were subjected to uniform compressive strain at the speed of 0.1 mm/s until the measured strain reached the value of −0.5 (see Figure 1c). In the cyclic straining tests, the sinusoidal waveform, shown in Figure 1b, was generated by using an average crosshead speed of 30 mm/s. Furthermore, five different controlled mean strain levels, $e_{mean}$, were considered, ranging between −6.76% ($\Delta_{c,mean}=-3.75\;\mathrm{mm}$) and −27.03% ($\Delta_{c,mean}=-15.0\;\mathrm{mm}$). In every cyclic test, 400 to 500 stress-strain data points were collected per cycle to construct the corresponding hysteresis loop. All of the monotonic tests and the cyclic tests were carried out by using a servo-hydraulic mechanical testing system and the load-displacement data were collected by using “Max” software (Version 7.0, Instron) integrated with the testing system.

## 3. Experimental Results and Observations

### 3.1. Monotonic Compression 

At the beginning of the compression, it was observed that the lateral deformation occurred on the tested rubber fender. The phenomenon of the lateral deformation became clear gradually due to the increasing compressive displacement. The lateral deformation is caused by high Poisson’s ratio of the rubber, and was illustrated in Figure 1c. The cylindrical rubber fender investigated in this study can transmit the compressive impact force between the vessel and the fender to the dock. Therefore, the compressive force in the compression test was called the transmission load in this paper.

Moreover, it is interesting to study the correlation between the transmission load and the compressive deformation of the tested rubber fender subjected to monotonic compression. For convenience, in this study, the transmission stress $S_{c}$ was defined as the ratio of the absolute value of the measured transmission load P to the original cross-sectional area of the damper $A_{o}$, i.e.,

(1) $$S_{c}=\left|P\right|/A_{o}.$$

Similarly, the compressive strain $e_{c}$ was evaluated by

(2) $$e_{c}=\left|\Delta_{c}\right|/H_{o},$$

where $\Delta_{c}$ was the measured compressive displacement and $H_{o}$ was the original height of the fender. 

**Figure 2:**
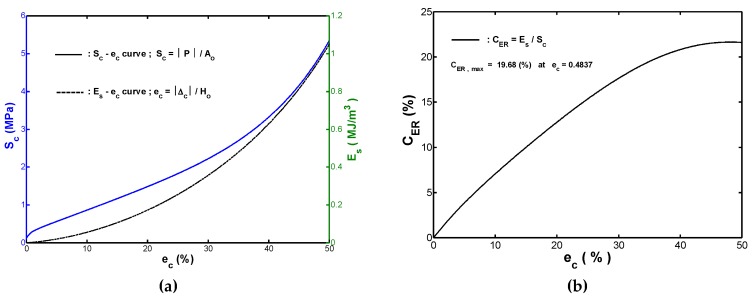
Experimental results of monotonic compression testing: (**a**) $S_{c}-e_{c}$ and $E_{s}-e_{c}$ curves, and (**b**) $C_{ER}-e_{c}$ curve.

The variation of the transmission stress $S_{c}$ with the compressive strain $e_{c}$ was plotted and shown in Figure 2a. The strain-energy-density was calculated by the following integration:

(3) $$E_{s}=\int_{0}^{e_{c,p}}S_{c}de_{c}.$$

It was clear that the area under the $S_{c}-e_{c}$ curve measured from $e_{c}=0$ to $e_{c}=e_{c,p}$ represented the absorbed strain energy per unit volume as the fender was subjected to a compressive strain $e_{c,p}$. Therefore, the value of $E_{s}$ corresponding to any strain value in the strain range $e_{c}=0\sim 0.5$ could be obtained via Equation (3). Similarly, the calculated values of $E_{s}$ and their corresponding strain $e_{c}$ could then be plotted, as shown in Figure 2a. The values of $E_{s}$ and $S_{c}$ at the maximum strain ($e_{c}=0.5)$ were denoted as the design compressive toughness $E_{s,c}$ and the design compressive strength $S_{c,c}$ of the fender, respectively. The experimental results revealed that the design compressive toughness $E_{s,c}$ was approximately $1.1359\;\mathrm{MJ}/m^{3},$ while the design compressive strength $S_{c,c}$ was 5.81 MPa. Figure 2a showed that the measured $S_{c}-e_{c}$ curve comprised two distinct regions. At low strain range (e.g., $e_{c}$ < 0.2), the transmission stress $S_{c}$ increased slowly with the increment of strain $e_{c}$. The slope of the measured $S_{c}-e_{c}$ curve decreased as the $e_{c}$ value was increased. This was because the lateral deformation has just been developed and was increased with the increment of compressive displacement from the beginning of the compression. Subsequently, it was found that the transmission stress increased more rapidly as the compressive strain was furtherly increased. This was due to the fact that the inner self-contact deformation strengthened the overall axial stiffness. In contrast, the absorbed strain-energy-density $E_{s}$ curve increased monotonically with the increment of the strain over the entire strain range ($e_{c}=0\sim 0.5)$. 

The energy absorption capacity of rubber fenders under the designed transmission force is a key consideration. In most applications, rubber fenders which provide a high energy absorption capacity at a specified value of the transmission force are preferred than those which do not. Consequently, for monotonic compressive loads, the performance of the fender could be quantified via the static effectiveness index $C_{ER}$, which was defined as the ratio of the absorbed strain-energy-density to the corresponding transmission stress, i.e.,

(4) $$C_{ER}=E_{s}/S_{c}$$

Figure 2b showed the $C_{ER}-e_{c}$ curve (referred to as the static performance-strain curve hereafter) obtained by substituting the $\left(S_{c},E_{s}\right)$ data at the corresponding compressive strain $e_{c}$ in Figure 2a into Equation (4). As shown in Figure 2b, $C_{ER}$ increased with the strain rise up to approximately $e_{c}=0.4837$ and then reduced very slightly to the final recorded strain ($e_{c}=0.5$). In other words, the optimal fender performance was obtained at the strain of $e_{c}=0.4837$ rather than at the final recorded strain. The value of $e_{c}$ corresponding to the best (i.e., highest) static effectiveness index was referred to as the rated strain hereafter. The fact that the rated strain was less than the final recoded strain implied that the increasing rate of $E_{s}$ at $e_{c}>0.4837$ was less than that for $S_{c}$. The inspection of the $C_{ER}-e_{c}$ curve in Figure 2b showed that $C_{ER}$ was 19.68% at the rated strain. In practice, the static effectiveness index at a specified value of $e_{c}$ provides a useful method for comparing the performance of fenders made from different rubber raw materials, in which a higher value of $C_{ER}$ indicates that a fender has a greater strain-energy-density absorption capacity under the same transmission stress than a fender with a lower value of $C_{ER}$ does. 

### 3.2. Cyclic Compression 

**Figure 3:**
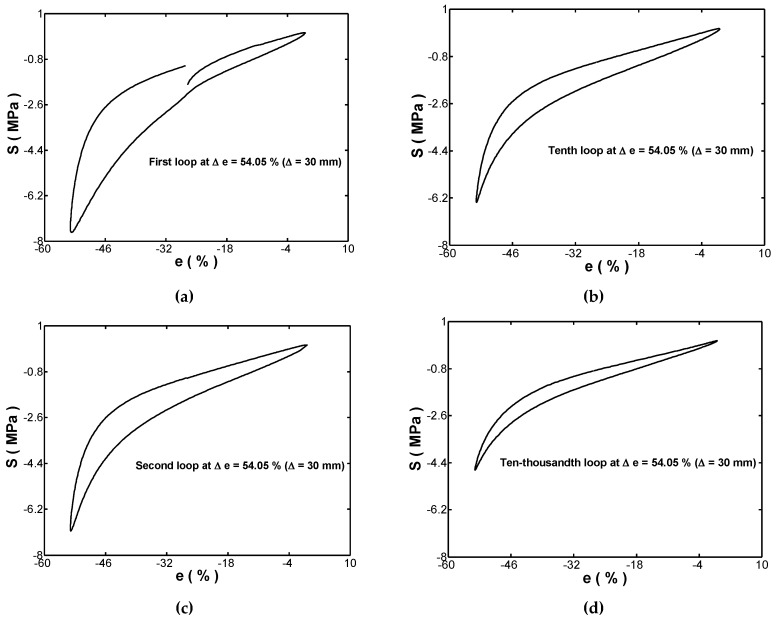
Cyclic hysteresis loops for: (**a**) first, (**b**) second, (**c**) tenth, and (**d**) ten-thousandth cycles in cyclic compression testing with $A_{e}=-1$ and strain range of 54.05 %.

Figure 3 showed the stress-strain hysteresis loops obtained in the first, second, tenth, and ten-thousandth cycles of the cyclic compression test performed with an amplitude ratio of $A_{e}=-1$ and a strain range of Δe=54.05% ($\mathrm{which}\;\mathrm{was}\;\mathrm{equal}\;\mathrm{to}\;\left|\Delta_{c}\right|=30\;\mathrm{mm}$). It was seen in Figure 3 that all of the hysteresis loops are closed except for the one obtained in the first cycle. In addition, the hysteresis loops shift progressively to a lower strain and higher stress, as the number of compression cycles increases. Finally, the stress range decreases significantly during the first several cycles and then is stabilized after an amount of applied cycles. This phenomenon is well-known as Mullins’ effect. 

In the cyclic compression tests ($A_{e}=-1$), the maximum transmission stress was zero and the minimum transmission stress occurred in the minimum strain condition. Hence, the magnitude of stress range ΔS was equal to the absolute value of the minimum transmitted stress.

**Figure 4:**
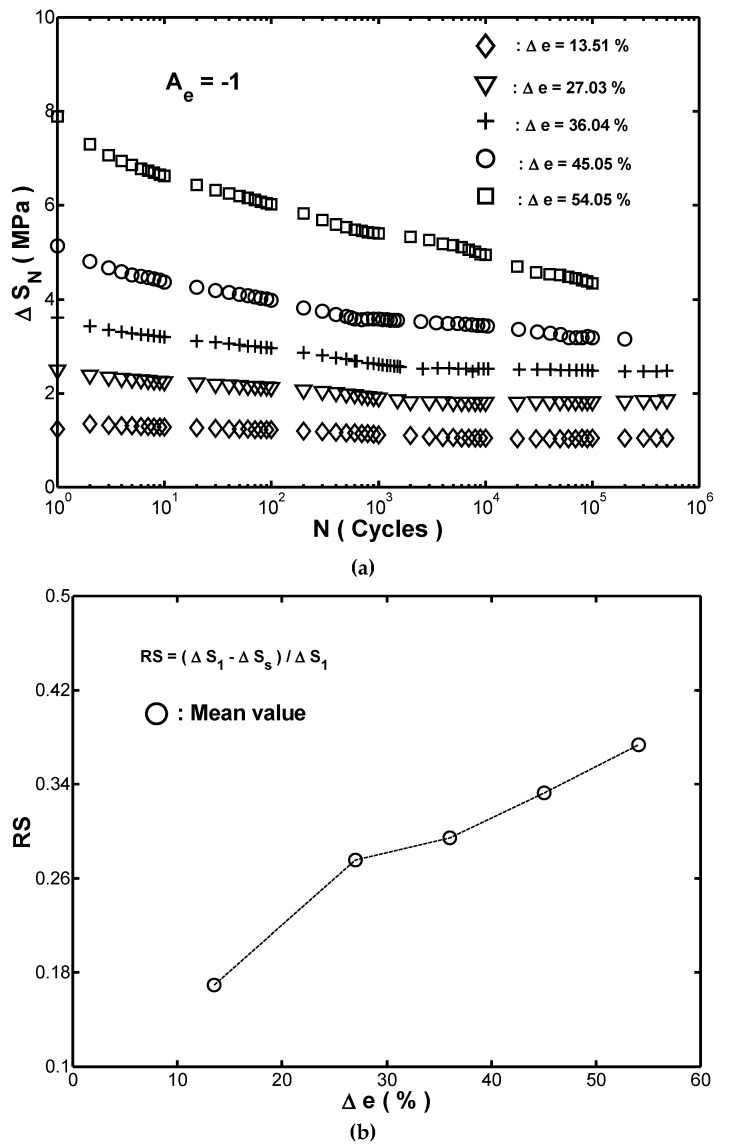
(**a**) Relationship between stress range ($\Delta S_{N}$) and number of cycles (N) in cyclic compression testing with five different strain ranges. Note that amplitude ratio was $A_{e}=-1$ in every case. (**b**) Variation of softening factor RS with strain range Δe in cyclic compression testing.

$\Delta S_{N}$ was the magnitude of the stress range at the *N*-th cycle. For the five strain ranges considered in this study, the variations of $\Delta S_{N}$ with the applied cycles N were shown in Figure 4a. For each of the five considered values of Δe, the magnitude of $\Delta S_{N}$ decreased progressively as the number of cycles increased toward ten thousand cycles and then remained approximately constant thereafter. In other words, the fender underwent a softening effect during the initial stages of cyclic straining. This result may be attributed to the fact that the applied cyclic straining as reported also in References [16,17] lead to the occurrence of the fracture in the carbon chain or weak bonds between the rubber and carbon. Based on the results shown in Figure 4a, the stress-strain hysteresis loops obtained in the ten-thousandth cycle were thus used to represent the stable behavior of the rubber fender. 

A close inspection of Figure 4a showed that the reduction of $\Delta S_{N}$ from the first cycle to the ten-thousandth cycle increased with an increasing strain range. In other words, the stress softening phenomenon was sensitive to the magnitude of the cyclic strain. To quantify the effect of the applied strain range on the cyclic stress softening, the following softening factor RS was introduced:

(5) $$RS=\frac{\left(\Delta S_{1}-\Delta S_{s}\right)}{\Delta S_{1}},$$

where $\Delta S_{1}$ was the stress range in the first cycle and $\Delta S_{s}$ was the stress range in the ten-thousandth cycle. Figure 4b showed the value of RS for each of the five considered cyclic strain ranges. It was found that RS increased dramatically as Δe was increased from 13.51% to 27.03%, but then its increasing trend was lowered as Δe was further increased to 54.05%. In other words, the value of RS increased as the strain range increased. This finding indicated that the cyclic stress softening phenomenon increased obviously with an increasing strain range. In the case of every loop, shown in Figure 3, the upper branch showed the correlation between the stress and the strain during the unloading process since the controlled strain was from −54.05% to zero, while the lower branch showed the stress-strain response during the loading process. $E_{s,r}$ represented the released strain-energy-density in unloading path, and it was equal to the area under the upper branch curve between e=−54.05% to $e=e_{1}$. Similarly, the symbol $E_{s,a}$ represented the absorbed strain-energy-density in loading path. Clearly, the stored strain-energy-density ($E_{s,a}-E_{s,r}$) in the fender was equal to the area enclosed within the hysteresis loop. During the cyclic loading, the accumulated strain-energy-density yielded the occurrence of permanent damage in the fender.

**Figure 5:**
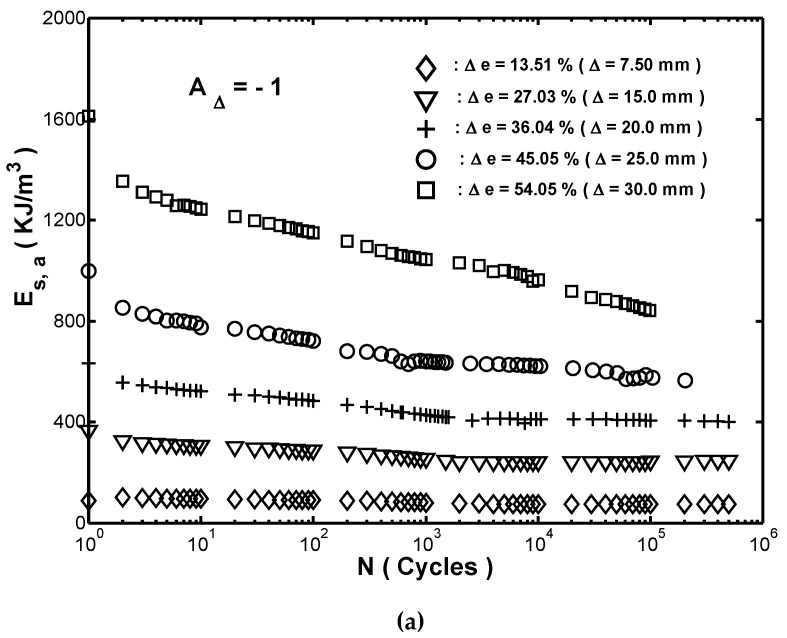

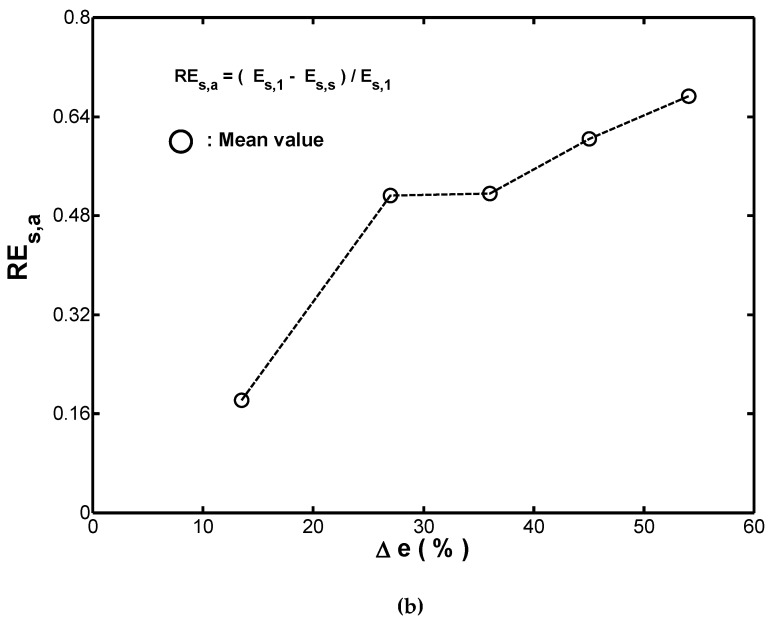
(**a**) Relationship between strain-energy-density absorption ($E_{s,a}$) and number of cycles (N) in cyclic compression testing with five different strain ranges. Note that amplitude ratio was $A_{e}=-1$ in every case. (**b**) Variation of energy reduction factor $RE_{s,a}$ with strain range Δe in cyclic compression testing.

To ensure the dock and the vessel can be protected by the fender during impact in berthing, the ability of the fender to absorb strain-energy-density is concerned during cyclic compression loading. Figure 5a showed a plot of the absorbed strain-energy-density $E_{s,a}$ with the number of cycles N at the five different strain ranges considered for the tested fender. It was seen that for every strain range, $E_{s,a}$ decreased with an increasing number of cycles over the first ten thousand cycles and then remained approximately constant. Moreover, the extent of the reduction in $E_{s,a}$ depended on the magnitude of the strain. To quantify the effect of the strain range on the reduction in $E_{s,a}$, the following energy reduction factor $RE_{s,a}$ was introduced:

(6) $$RE_{s,a}=\left(E_{s,a1}-E_{s,as}\right)/E_{s,a1}\;,$$

where both subscripts “1” and “s” in Equation (6) denoted the first cycle and the ten-thousandth cycle, respectively. Figure 5b showed the calculated value of $RE_{s,a}$ for each of the five considered strain ranges. It was seen that the variation of $RE_{s,a}$ with Δe was similar to that of RS with Δe (see Figure 4b). In other words, the amount by which the absorbed strain-energy-density reduces with an increasing number of cycles. Comparing the $RE_{s,a}-\Delta e$ curve in Figure 5b with the *RS* −Δe curve in Figure 4b, it was found that the extent of the reduction in $E_{s,a}$ for a specified strain range was greater than that in ΔS. Thus, to properly quantify the performance of the rubber fender during cyclic straining, the named cyclic effectiveness index was also proposed in this study. In contrast to the $C_{ER}$ index, the following cyclic effectiveness index $DC_{ER}$ was introduced:

(7) $$DC_{ER}=\;E_{s,a}/\Delta S\;.$$

**Figure 6:**
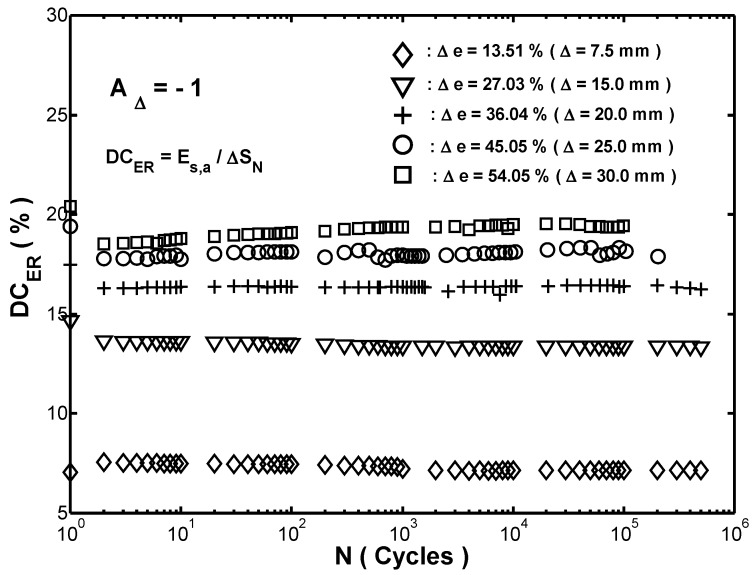
Relationship between cyclic effectiveness index ($DC_{ER}$) and number of cycles (N) in cyclic compression testing with five different strain ranges.

$DC_{ER}$ was a useful parameter for comparing the strain-energy-density absorption capacities of different fenders at specific value of ΔS. With the help of ΔS−N data, shown in Figure 4a, and $E_{s,a}-N$ data, shown in Figure 5a, the value of $DC_{ER}$ was calculated and then plotted against the corresponding number of cycles in Figure 6. As shown in Figure 6, it was seen that the values of $DC_{ER}$ at the five strain ranges were changed drastically during the first two cycles and then remained approximately constant thereafter; the value of $DC_{ER}$ increased significantly with an increasing strain range at a specified number of cycles.

### 3.3. Stable Cyclic Response 

**Figure 7:**
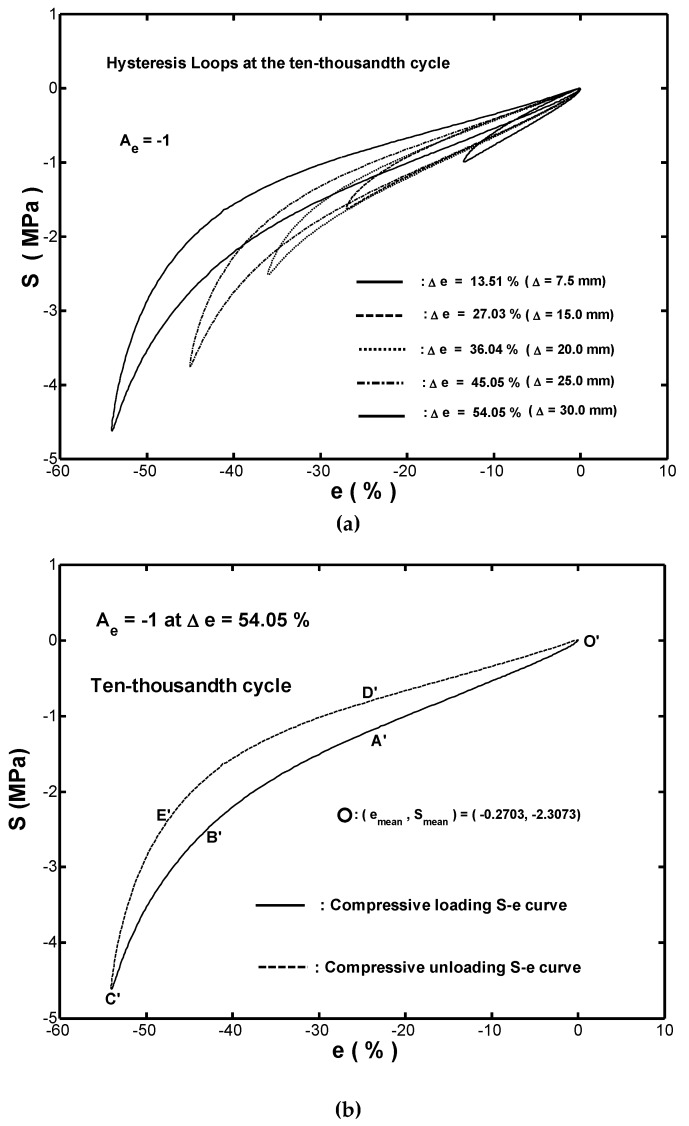
(**a**) Stable hysteresis loops in cyclic compression testing with five different strain ranges (Δe=13.51%,27.03%,36.04%,45.05%, and 54.05%). (**b**) Upper and lower branches of stable hysteresis loop for strain range of Δe=54.05%.

Figure 7 showed the stress-strain hysteresis loops obtained in the ten-thousandth cycles of the cyclic compressive tests performed under the five different strain ranges. Neither the upper nor the lower branches of the hysteresis loops overlapped. The observation confirmed that the Massing cyclic stress-strain behavior [18] was absent for the present rubber fender. 

Figure 7b presented an enlarged view of the hysteresis loop in Figure 7a for a strain range of $\Delta e=54.05\%\;(\Delta_{a}\;=30\;\mathrm{mm}$). Obviously, a different response in upper and lower branches was observed. A similar result was found for all of the hysteresis loops, shown in Figure 7a. Thus, all of the loops had an asymmetric behavior. Notably, the fact that the stable stress-strain responses for the fender were asymmetric behavior in compressive loading and compressive unloading implied that two different stress-strain relations were required to model the upper and lower branches, respectively. 

## 4. Modeling and Discussions

### 4.1. Modeling Stress-Strain Curve in Monotonic Compression 

Generally speaking, polynomial regression is a simple and easy approach to model the relationship between dependent variable and independent variable in the experimental analysis. Notably, polynomial regression is adequate to fit a monotonic response curve. In practice, the accuracy of prediction is highly dependent on the degree of the polynomial regression. However, in engineering applications, it is usually desirable to minimize the degree of the polynomials for computational simplicity. Thus, the minimum degree of the polynomial function which achieves the best fit between the fitted curve and the experimental data is generally estimated via a simple process of trial and error. 

As shown in Figure 2a, for the presented monotonic compression tests, the absorbed strain-energy-density $E_{s}$ increased monotonically with the increment of strain over the range of $e_{c}=0\sim 0.5$. Therefore, a new mathematical model for strain-energy-density function expressed in terms of the variable $e_{c}$, $E_{s}\left(e_{c}\right)$, was proposed and modeled as a polynomial with n degree in this study. Moreover, polynomial regression coefficients are determined by using ordinary least-square estimation. The energy-polynomial-function $E_{s}\left(e_{c}\right)$ by performing a polynomial regression on the measured ($e_{c},E_{s})$ data shown in Figure 2a was given as:

(8) $$E_{s}=C_{4}\times e_{c}^{4}+C_{3}\times e_{c}^{3}+C_{2}\times e_{c}^{2}+C_{1}\times e_{c}+C_{o}.$$

**Figure 8:**
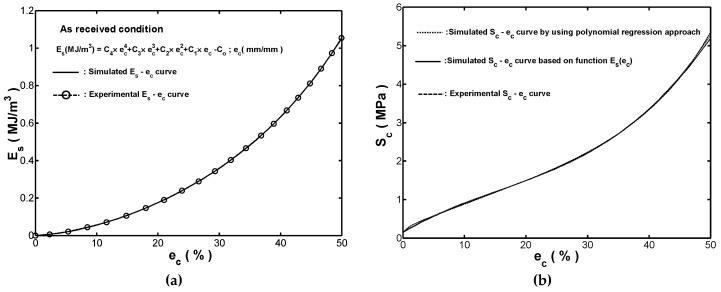
Comparison of experimental results and simulation results based on polynomial functions for (**a**) $E_{s}-e_{c}$ curve, and (**b**) $S_{c}-e_{c}$ curve.

In Equation (8), the five polynomial regression coefficients determined were $C_{4}=11.9613$, $C_{3}=-7.4198$, $C_{2}=4.6297$, $C_{1}=0.1503$ and $C_{o}=0.001$, respectively, and $E_{s}$ was measured with the unit of MJ/$m^{3}$. By using a polynomial regression on the measured ($e_{c},E_{s})$ data shown in Figure 2a, Figure 8a compared the measured $E_{s}-e_{c}$ curve and the fitted one obtained by using Equation (8). It was seen that the two sets of results were in excellent agreement. Based on the observation, it was confirmed that a 4th-degree polynomial $E_{s}\left(e_{c}\right)$ was enough to provide a highly accurate result in the description of the $E_{s}-e_{c}$ curve in compression for the tested fender. 

Recalling that the magnitude of $E_{s}$ for the fender was obtained by determining the area under the $S_{c}-e_{c}$ curve via integration, which implied that $E_{s}$ and $S_{c}$ were related as:

(9) $$S_{c}=dE_{s}/de_{c}.$$

In other words, the transmission stress $S_{c}$ for a given compressive strain was determined from the differentiation of energy-polynomial-function $E_{s}\left(e_{c}\right)$ with respect to the compressive strain. For the tested fender, the stress-polynomial-function $S_{c}\left(e_{c}\right)$ was found to be:

(10) $$S_{c}=d_{3}\times e_{c}^{3}+d_{2}\times e_{c}^{2}+d_{1}\times e_{c}+d_{o},$$

where $d_{3}=4\times C_{4}$, $d_{2}=3\times C_{3}$, $d_{1}=2\times C_{2}$, and $d_{o}=C_{1}$. Figure 8b compared the measured $S_{s}-e_{c}$ curve (reproduced from Figure 2a) with the one fitted by using Equation (10). A good agreement between the two curves was once again observed. In other words, the validity of the polynomial functions given in Equations (8) and (10) is confirmed.

### 4.2. Modeling the Stable Stress-Strain Hysteresis Loop Curve 

**Figure 9:**
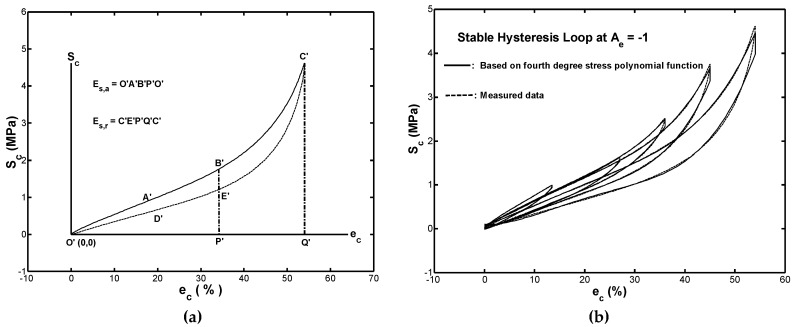

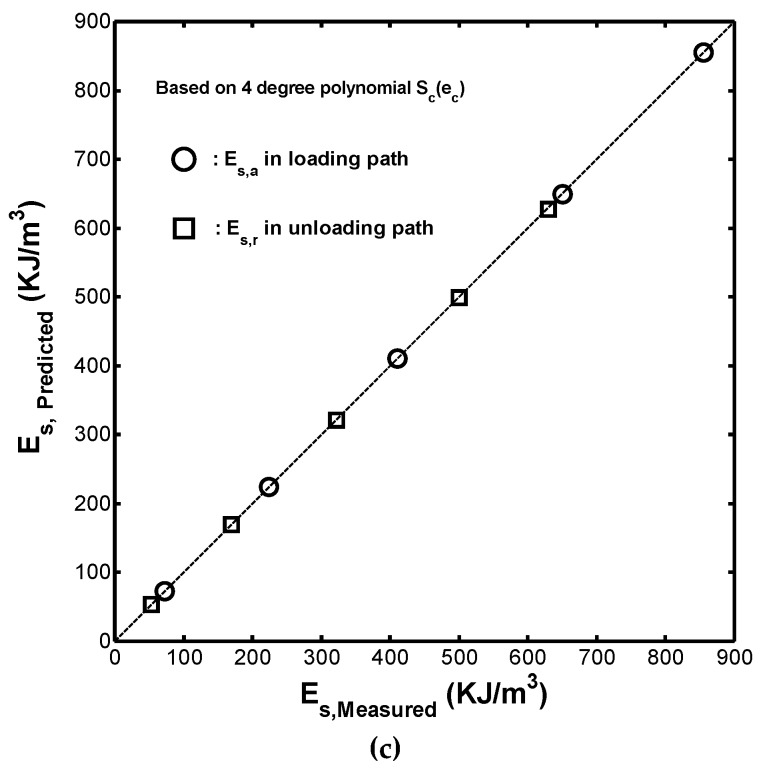
(**a**) Illustration of absorbed strain-energy-density $E_{s,a}$ and released strain-energy-density $E_{s,r}$, (**b**) comparison of simulated and measured hysteresis loops, and (**c**) comparison of calculated and measured values of $E_{s,a}$ and $E_{s,r}$ for various strain ranges.

As shown in Figure 8b, the stress function given in Equation (10) provided a highly accurate description of the relationship between the transmission stress $S_{c}$ and the compressive strain $e_{c}$ in monotonic compression. Thus, it was reasonable to infer that all stable hysteresis loops shown in Figure 7a obtained under cyclic compression could also be simulated by the stress polynomial function, $S_{c}\left(e_{c}\right)$. As described in Section 3.3, the hysteresis loops were asymmetric about their respective centers, so that two stress-strain relations were required to describe the upper and lower branches, respectively. Figure 9a plotted the hysteresis loop shown in Figure 7b for a strain range of Δe=54.05% in the absolute-value form. Hence, the upper branch of the loop (i.e., $O^{\prime }A^{\prime }B^{\prime }C^{\prime }$) corresponded to the loading path, while the lower branch (i.e., $C^{\prime }E^{\prime }D^{\prime }O^{\prime }$) corresponded to the unloading path. Similarly, both symbols $E_{s,a}$ and $E_{s,r}$ would be used to represent the strain-energy-density in loading and unloading path at a specified strain, $e_{c}$, respectively. 

Similar to the development of energy-polynomial-functions $E_{s}\left(e_{c}\right)$ in monotonic loading condition, the energy-polynomial-function $E_{s}{\left(e_{c}\right)}_{a}$ corresponding to every upper branch curve shown in Figure 9b was determined by performing a polynomial regression on the measured energy data with the corresponding strain data. Subsequently, the energy-polynomial-functions $E_{s}{\left(e_{c}\right)}_{r}$ corresponding to every lower branch curve was also determined. Notably, the least square estimation was applied to determine the polynomial regression coefficients for all energy-polynomial-functions in loading/unloading conditions. In other words, polynomial regression was used to model the relationship between the dependent variable $e_{c}$ and the independent variable $E_{s,a}$($E_{s,r}$) for every measured hysteresis loop shown in Figure 9b. In the monotonic modeling, via a trial-and-error on the least-square fitting approach, it was found that a 5th-degree polynomial in $e_{c}$ provided a good fit with the measured $\left(e_{c},\;E_{s,a}\right)$ data and $\left(e_{c},\;E_{s,r}\right)$ data for the stable hysteresis loops (in absolute-value form) at the five considered strain ranges shown in Figure 9b. Table 2 listed the polynomial regression coefficients of a 5-degree polynomial for all modeled energy-polynomial-functions in loading path, $E_{s}{\left(e_{c}\right)}_{a}$, and in unloading path, $E_{s}{\left(e_{c}\right)}_{r}$.

In the monotonic case, the stress-polynomial-function $S_{c}\left(e_{c}\right)$ for the loading path of every cyclic compression test could be obtained by differentiating the energy-polynomial-function $E_{s}{\left(e_{c}\right)}_{a}$ with respect to the variable $e_{c}$. Similarly, the stress-polynomial-function $S_{c}\left(e_{c}\right)$ in the unloading path was also obtained in the supply of the energy-polynomial-function $E_{s}{\left(e_{c}\right)}_{r}$. Figure 9b compared the simulated hysteresis loops obtained by using these developed energy-polynomial-functions with the measured hysteresis loops. In general, a good agreement was found between the two sets of results for every loop. Figure 9c compared the calculated and measured strain-energy-density absorption for every measured hysteresis loop, shown in Figure 9b. It was observed that most of the plotted points fall along the diagonal line (i.e., the line of perfect correlation). In other words, the 4-degree stress-polynomial-function $S_{c}\left(e_{c}\right)$ provided an extremely accurate estimation of the absorption/resilience strain-energy-density property of the tested rubber fender. 

### 4.3. Development of Cyclic Stress-Strain Curve and Cyclic Energy-Strain Curve

**Figure 10:**
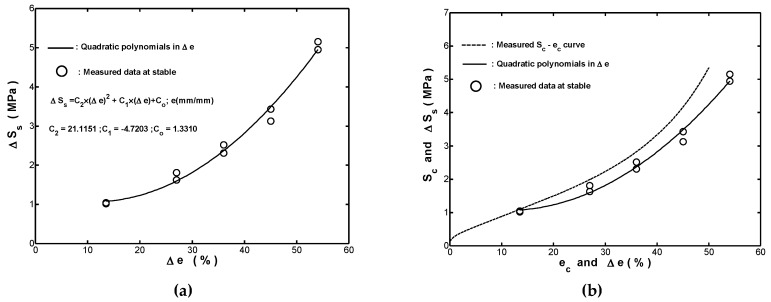
(**a**) Comparison of fitted $\Delta S_{s}-\Delta e$ curve and measured data ($\Delta e,\;\Delta S_{s}$ ) for the ten-thousandth cycle of cyclic straining test performed with $A_{e}=-1$, and (**b**) comparison of measured $S_{c}-e_{c}$ curve and fitted $\Delta S_{s}-\Delta e$ curve.

In general, the cyclic stress-strain curve obtained by plotting the stable stress range $\Delta S_{s}$ with the corresponding strain range Δe provides much useful information related to the deformation behavior of structures and components subjected to cyclic loading. For the fender considered in this study, an inspection of the measured $\left(\Delta e,\;\Delta S_{s}\right)$ data in Figure 4a showed that the $\Delta S_{s}-\Delta e$ curve could be estimated by the following quadratic polynomial

(11) $$\Delta S_{s}\left(\mathrm{MPa}\right)=21.1151\times {\left(\Delta e\right)}^{2}-4.7203\times \left(\Delta e\right)+1.3310;\;\Delta e\;\left(\mathrm{mm}/\mathrm{mm}\right)$$

where the polynomial regression coefficients in Equation (11) were obtained by using a least-square estimation. Figure 10a showed that the fitted $\Delta S_{s}-\Delta e$ curve obtained by using Equation (11) was in good agreement with the measured $\left(\Delta e,\;\Delta S_{s}\right)$ data. Figure 10b compared the measured monotonic compressive stress-strain curve and the fitted one obtained by using Equation (11). It was seen that the monotonic $S_{c}-e_{c}$ curve lies above the fitted $\Delta S_{s}-\Delta e$ curve, which revealed that the fender undergoes a softening effect during long-term cyclic straining. 

By performing a polynomial regression on the measured stable strain-energy-density in loading $E_{s,as}$ with respect to the applied strain range Δe, the relationship between $E_{s,as}$ and Δe were found by the following cubic polynomial:

(12) $$E_{s,as}\left(\mathrm{KJ}/m^{3}\right)=10422.4\times {\left(\Delta e\right)}^{3}-6190.2\times {\left(\Delta e\right)}^{2}+2424.7\times \left(\Delta e\right)-166.63;\;\Delta e\;\left(\mathrm{mm}/\mathrm{mm}\right)$$

**Figure 11:**
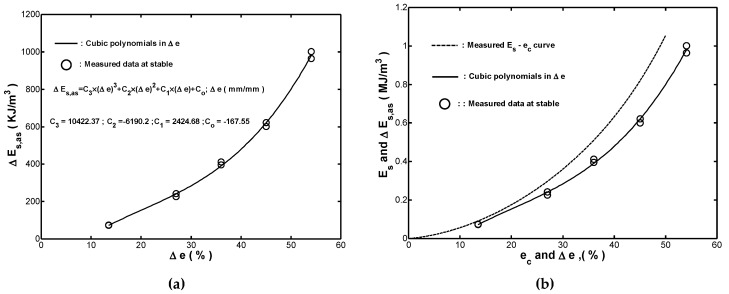
(**a**) Comparison of fitted $E_{s,as}-\Delta e$ curve and ($\Delta e,\;E_{s,s})$ data at stable for cyclic straining test performed with $A_{e}=-1$, and (**b**) comparison of measured $E_{s}-e_{c}$ curve and fitted $E_{s,as}-\Delta e$ curve.

As shown in Figure 11a, the fitted cyclic energy-strain curve $E_{s,as}-\Delta e$ obtained by using Equation (12) was in good agreement with the measured ($\Delta e,E_{s,as}$) data. In design, the cyclic energy-strain curve could provide a proper estimation of the strain-energy-density absorption for the cylindrical rubber fender under cyclic compressive loading. Figure 11b compared the measured $E_{s}-e_{c}$ curve with the fitted $E_{s,as}-\Delta e$ curve. It was seen that the fitted $E_{s,as}-\Delta e$ curve was located below the measured $E_{s}-e_{c}$ curve. Based on the observation in Figure 11b, it was again confirmed that the cyclic straining reduced the energy absorption capability of the tested fender, particularly at higher strains. Notably, the results presented in Figure 11b indicated that the safety of a structure protected by a fender could not be guaranteed if the monotonic $E_{s}-e_{c}$ curve was used to determine the designed values of the strain-energy-density absorption capacity of the fender during the ship berthing. Instead, the fender should be designed by considering the fitted $E_{s,as}-\Delta e$ curve obtained under cyclic loading. 

As described in Section 3.2, the $DC_{ER}$ (see Equation (7)) was a useful parameter for comparing the cyclic strain-energy-density absorption capacities of different fenders at specific values of ΔS and *N*. For convenience, the cyclic $DC_{ER}$ corresponding to the ten-thousandth cycle was denoted as $DC_{ER,S}$ and the corresponding $DC_{ER,S}-\Delta e$ curve was referred to as the cyclic performance-strain curve. From an inspection of the ($\Delta e,\;\Delta S_{s}$) and ($\Delta e,\;E_{s,as}$) data shown in Figure 10a and Figure 11a, respectively, the $DC_{ER,S}-\Delta e$ curve could be fitted by using a cubic polynomial as follows:

(13) $$DC_{ER,S}=-0.231\times {\left(\Delta e\right)}^{3}+0.4311\times {\left(\Delta e\right)}^{2}+0.6826\times \left(\Delta e\right)-0.0119;\;\Delta e\left(\mathrm{mm}/\mathrm{mm}\right),$$

**Figure 12:**
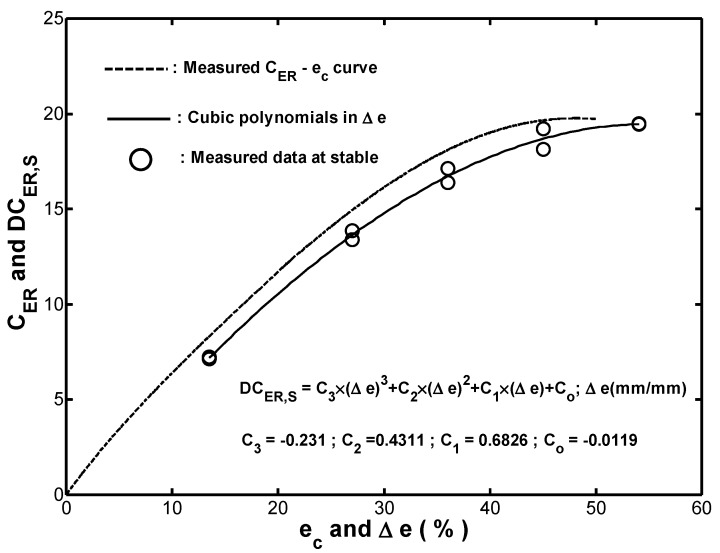
Comparison of measured $C_{ER}-e_{c}$ curve, ($\Delta e,DC_{ER,S}$ ) data at stable, and fitted $DC_{ER,S}-\Delta e$ curve.

As shown in Figure 12, it was seen that the measured $C_{ER}-e_{c}$ curve obtained in monotonic compression was above the fitted $DC_{ER,S}-\Delta e$ curve for cyclic compression. In other words, cyclic straining reduced the performance of the fender.

## 5. Conclusions 

This study presented an experimental investigation on the monotonic and cyclic deformation behavior of a cylindrical rubber fender in compression. The experimental observations and analyses supported the following main conclusions:

1. The lateral deformation occurred in the tested rubber fender and became significant gradually due to the increasing compressive displacement.
2. The measured values of $S_{c}$ and $E_{s}$ under monotonic compression increased with the increment of $e_{c}$ up to the final recorded strain of $e_{c}=0.5$. The maximum ratio of $E_{s}$ to $S_{c}$ appeared at a slightly lower strain than the final recorded one.
3. In cyclic compression testing, the stress-strain hysteresis loops were all closed with the exception of that obtained from the first cycle.
4. The stress-strain hysteresis loops obtained in the ten-thousandth cycle were taken to represent the stable behavior of the tested rubber fender under cyclic loading.
5. This Mullins’ effect is observed in the cyclic behavior of the tested rubber fender under cyclic compression.
6. Both properties RS and $RE_{s,a}$ were introduced to quantify the effect of the strain range Δe on the extent of the reduction in $\Delta S_{N}$ and $E_{s,a}$. It was found that the calculated values of RS and $RE_{s,a}$ increased with an increasing strain range.
7. Massing cyclic stress-strain behavior is absent in the stable cyclic response of the tested fender.
8. A new expression based on polynomial function for strain-energy-density function $E_{s}\left(e_{c}\right)$ was presented to simulate the monotonic and cyclic stable stress-strain response of the tested rubber fender in compression. The stress-polynomial-function $S_{s}(e_{c}$) for the tested fender under compression was determined by differentiating the proposed energy-polynomial-function $E_{s}\left(e_{c}\right)$ with respect to the strain $e_{c}$. Regression coefficients in $E_{s}\left(e_{c}\right)$ could be obtained by using ordinary least-square estimation to the measured data.
9. Since the presented stress-polynomial-function $S_{s}(e_{c}$) provided good fits to the monotonic compression curve and the shape of the stable hysteresis loop under different strain ranges, the validity of the proposed approach was confirmed.
10. Cyclic stress-strain curve and cyclic energy-strain curve were developed and also modeled to provide a proper estimation of the mechanical properties of cylindrical rubber fender under compressive loading.

## Figures and Tables

**Table 1:** Composition of SBR/NR hybrid vulcanized rubber used in this study.

Sample Ingredients	Quantity (phr)
Natural rubber	50.0
Styrene butadiene rubber (SBR)	50.0
Zinc oxide	5.0
Aging agent (4020)	1.5
Aging agent (RD)	1.5
Microcrystalline wax	1.0
Resin	1.5
HAF(high abrasion furnace ) carbon blacks	45
Aromatic oil	5.0
Sulfur	2.5
Promoter (DM)	0.65

phr, parts per hundred rubber.

**Table 2:** Polynomial regression coefficients for $E_{s,a}\left(e_{c}\right)$ and $E_{s,r}\left(e_{c}\right)$.

$E_{s,a}\left(MJ/m^{3}\right)=C_{5}\times e_{c}{}^{5}+C_{4}\times e_{c}{}^{4}+C_{3}\times e_{c}{}^{3}+C_{2}\times e_{c}{}^{2}+C_{1}\times e_{c}+C_{o}\;;\;e_{c}\;\left(\;\mathrm{mm}/\mathrm{mm}\right)$						
$\Delta e_{c}\;\left(\%\right)$	$C_{5}$	$C_{4}$	$C_{3}$	$C_{2}$	$C_{1}$	$C_{o}$
54.05	21.8427	−18.6860	5.8263	1.6201	0.0910	−0.0006
45.05	27.9612	−18.6617	4.0173	2.4560	0.0760	−0.0004
36.04	20.4431	−8.3889	0.1439	3.0941	0.0544	−0.0002
27.03	−2.2424	9.1967	−4.3922	3.4920	0.0380	−0.0001
13.51	−316.4647	133.9596	−21.8165	5.0639	0.0247	−0.0000
$E_{s,r}\left(MJ/m^{3}\right)=C_{5}\times e_{c}{}^{5}+C_{4}\times e_{c}{}^{4}+C_{3}\times e_{c}{}^{3}+C_{2}\times e_{c}{}^{2}+C_{1}\times e_{c}+C_{o}\;;\;e_{c}\;\left(\;\mathrm{mm}/\mathrm{mm}\right)$						
$\Delta e_{c}\;\left(\%\right)$	$C_{5}$	$C_{4}$	$C_{3}$	$C_{2}$	$C_{1}$	$C_{o}$
54.05	37.1754	−36.6438	13.0661	−0.2676	0.1035	−0.0007
45.05	49.9004	−40.6625	12.2517	0.4867	0.0660	−0.0003
36.04	51.5481	−33.0275	8.0816	1.3910	0.0178	−0.0002
27.03	63.1038	−28.9018	5.5643	1.6915	−0.0016	−0.0000
13.51	493.2321	−124.1657	14.7223	2.0358	−0.0098	−0.0000

## Nomenclature

$S_{c}$	Transmission stress
$\Delta S_{N}$	Stress range at a specified cycle
$\Delta S_{s}$	Stress range in stable condition
$e_{c}$	Compressive strain
Δe	Strain range
$E_{s}$	The absorbed strain-energy-density during monotonic compression
$E_{s,a}$	The absorbed strain-energy- density during cyclic compression
$E_{s,r}$	The released strain-energy-density during cyclic compression
$C_{ER}$	Static effectiveness index
$DC_{ER}$	Cyclic effectiveness index
RS	Softening factor
$RE_{s,a}$	Energy reduction factor
$S_{c}\left(e_{c}\right)$	Stress-polynomial-function
$E_{s}\left(e_{c}\right)$	Energy-polynomial-function
